# Supplementary material for: Accurate model and ensemble refinement using cryo-electron microscopy maps and Bayesian inference
Source: PLoS Comput Biol. 2024 Jul 15;20(7):e1012180. doi: 10.1371/journal.pcbi.1012180 (PMC11271924; doi:10.1371/journal.pcbi.1012180)
Supplement: S1 Text — Table A. Parameters of the forward model to predict a density map from an atomistic model. These parameters are obtained from a 5-Gaussians fit of the electron atomic scattering factors for s up to 6.0 Å-1. B! parameters are expressed in Å2. Table B. Parameters of the forward model to predict a density map from a Martini 3 coarse grained model. B! parameters are expressed in Å2. Table C. Details of the systems used in the single-structure and ensemble refinement benchmark. For each system, the first 8 columns report details about the deposited structure: PDB and EMDB ids, resolution, number of protein residues, number of protein chains, number of resolved water molecules, number of resolved lipids, number of ligands. The last 5 columns contain details of the system as prepared for EMMIVox simulations: box size, number of water molecules, number of lipids, number of buffer ions, and total number of atoms. Fig A. The cryo-EM and cryo-ET revolutions. Number of (a) single-particle cryo-EM and (b) subtomogram averaging maps released per year and classified based on resolution. Data was obtained from the Electron Microscopy Data Bank (https://www.ebi.ac.uk/emdb/) on October 12th 2023. Fig B. Assessment of the quality of the cryo-EM structures deposited in the PDB database. Violin plots of (a) CC"#$!, (b) EMRinger score, (c) clashscore, and (d) MolProbity score as a function of the resolution of the cryo-EM structures. For this analysis, 2476 structures determined by single-particle cryo- EM in the resolution range 1.78 Å to 4.00 Å and released between 2015 and 2022 were used. In the violin plots, the white circle corresponds to the median value, the black rectangle extends from the first to the third quantiles, and the thin black line represents the 95% confidence intervals. Fig C. Benchmark of the approach to remove correlated voxels. Analysis of the number of voxels per atom for 2477 structures determined by single-particle cryo-EM between 2015 and 2022 in the resolutio [file pcbi.1012180.s001.pdf]

SUPPLEMENTARY INFORMATION

Accurate model and ensemble refinement using cryo-electron microscopy maps and Bayesian inference

S. E. Hoff[1,*], F. E. Thomasen[2], K. Lindorff-Larsen[2], M. Bonomi[1,*]

[1]Institut Pasteur, Université Paris Cité, CNRS UMR 3528, Computational Structural Biology Unit, Paris, France

[2]Structural Biology and NMR Laboratory, Linderstrøm-Lang Centre for Protein Science, Department of Biology, University of Copenhagen, DK-2200 Copenhagen, Denmark

*Corresponding authors: samuel.hoff@pasteur.fr, mbonomi@pasteur.fr

List of Supplementary Tables

Atom type	**A_1**	**A_2**	**A_3**	**A_4**	**A_5**	**B_1**	**B_2**	**B_3**	**B_4**	**B_5**
C	0.0489	0.2091	0.7537	1.142	0.3555	0.114	1.0825	5.4281	17.8811	51.134
O	0.0365	0.1729	0.5805	0.8814	0.3121	0.0652	0.6184	2.9449	9.6298	28.2194
N	0.0267	0.1328	0.5301	1.102	0.4215	0.0541	0.5165	2.8207	10.6297	34.3764
S	0.0915	0.4312	1.0847	2.4671	1.0852	0.0838	0.7788	4.3462	15.5846	44.63655
P	0.1005	0.4615	1.0663	2.5854	1.2725	0.0977	0.9084	4.9654	18.5471	54.3648
F	0.0382	0.1822	0.5972	0.7707	0.213	0.0613	0.5753	2.6858	8.8214	25.6668
Na	0.126	0.6442	0.8893	1.8197	1.2988	0.1684	1.715	8.8386	50.8265	147.2073
Mg	0.113	0.5575	0.9046	2.158	1.4735	0.1356	1.3579	6.9255	32.3165	92.1138
Cl	0.0799	0.3891	1.0037	2.3332	1.0507	0.0694	0.6443	3.5351	12.5058	35.8633
Ca	0.2355	0.9916	2.3959	3.7252	2.5647	0.1742	1.8329	8.8407	47.4583	134.9613
K	0.2149	0.8703	2.4999	2.3591	3.0318	0.166	1.6906	8.7447	46.7825	165.6923
Zn	0.178	0.8096	1.6744	1.9499	1.4495	0.0876	0.865	3.8612	18.8726	64.7016

Table A. Parameters of the forward model to predict a density map from an atomistic model. These parameters are obtained from a 5-Gaussians fit of the electron atomic scattering factors for *s* up to 6.0 $Å^{-1}$. B_k parameters are expressed in $Å^2$.

Bead type	A_1	B_1	Bead type	A_1	B_1	Bead type	A_1	B_1	Bead type	A_1	B_1
ALA_BB	9.00	22.00	HIS_BB	9.50	23.00	ASN_BB	9.00	22.00	VAL_BB	9.50	23.00
ALA_SC1	0.50	0.50	HIS_SC1	4.50	11.50	ASN_SC1	9.00	18.50	VAL_SC1	7.00	18.00
CYS_BB	9.50	23.00	HIS_SC2	4.00	9.00	PRO_BB	9.50	23.50	TRP_BB	9.50	23.00
CYS_SC1	5.50	8.50	HIS_SC3	4.00	8.50	PRO_SC1	7.00	17.50	TRP_SC1	4.50	11.50
ASP_BB	9.50	23.00	ILE_BB	9.50	23.00	GLN_BB	9.00	22.00	TRP_SC2	4.00	9.00
ASP_SC1	8.50	17.00	ILE_SC1	10.00	25.50	GLN_SC1	11.50	24.50	TRP_SC3	4.50	11.00
GLU_BB	9.00	22.00	LYS_BB	9.00	22.00	ARG_BB	9.50	23.00	TRP_SC4	4.50	11.00
GLU_SC1	11.50	24.00	LYS_SC1	7.00	18.00	ARG_SC1	7.00	18.00	TRP_SC5	4.00	9.50
PHE_BB	9.50	23.00	LYS_SC2	4.50	11.00	ARG_SC2	9.00	18.00	TYR_BB	9.50	23.00
PHE_SC1	7.00	17.50	LEU_BB	9.00	22.00	SER_BB	9.50	23.00	TYR_SC1	4.50	12.00
PHE_SC2	4.50	11.00	LEU_SC1	9.50	21.50	SER_SC1	4.00	9.00	TYR_SC2	4.50	11.00
PHE_SC3	4.50	11.00	MET_BB	9.00	22.00	THR_BB	9.50	23.00	TYR_SC3	4.50	11.00
GLY_BB	9.50	23.00	MET_SC1	11.50	22.50	THR_SC1	7.00	17.00	TYR_SC4	4.00	8.50

Table B. Parameters of the forward model to predict a density map from a Martini 3 coarse grained model. B_k parameters are expressed in Å^2.

PDB id	EMDB id	res [Å]	# prot resid	# prot chains	# waters	# lipids	# ligands	box size [Å]	# waters	# lipids	# buffer ions	# atoms
7p6a	13223	1.90	540	5	76	0	0	122-70-48	10,782	0	124	40,805
7w9w	32377	2.02	810	3	144	24	0	90-90-113	17,548	169	113	86,452
7n00	24095	2.27	806	4	0	0	0	115-115-115	43,754	0	260	143,124
7t4n	25681	2.35	914	2	0	0	0	150-150-150	100,987	0	574	318,262
7b5o	12042	2.50	651	3	73	0	1	120-120-120	50,944	0	289	163,693
7mjs	23883	3.03	715	3	0	1	2	90-90-156	29,943	190	168	126,588
7lq6	23482	3.28	717	1	0	0	0	139-139-139	80,921	0	465	254,485
7nqk	12528	3.50	781	2	0	0	0	115-115-143	39,764	328	222	175,791
6yeg	10792	4.00	2064	12	0	0	0	132-132-132	60,592	0	496	212,980

Table C. Details of the systems used in the single-structure and ensemble refinement benchmark. For each system, the first 8 columns report details about the deposited structure: PDB and EMDB ids, resolution, number of protein residues, number of protein chains, number of resolved water molecules, number of resolved lipids, number of ligands. The last 5 columns contain details of the system as prepared for EMMIVox simulations: box size, number of water molecules, number of lipids, number of buffer ions, and total number of atoms.

List of Supplementary Figures

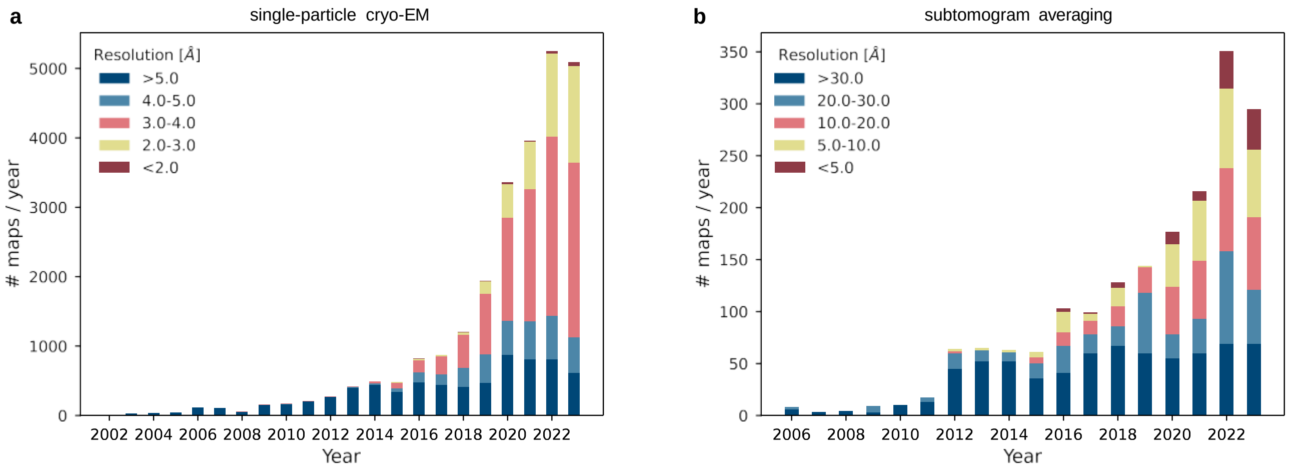

Fig A. The cryo-EM and cryo-ET revolutions. Number of (**a**) single-particle cryo-EM and (**b**) subtomogram averaging maps released per year and classified based on resolution. Data was obtained from the Electron Microscopy Data Bank (https://www.ebi.ac.uk/emdb/) on October 12th 2023.

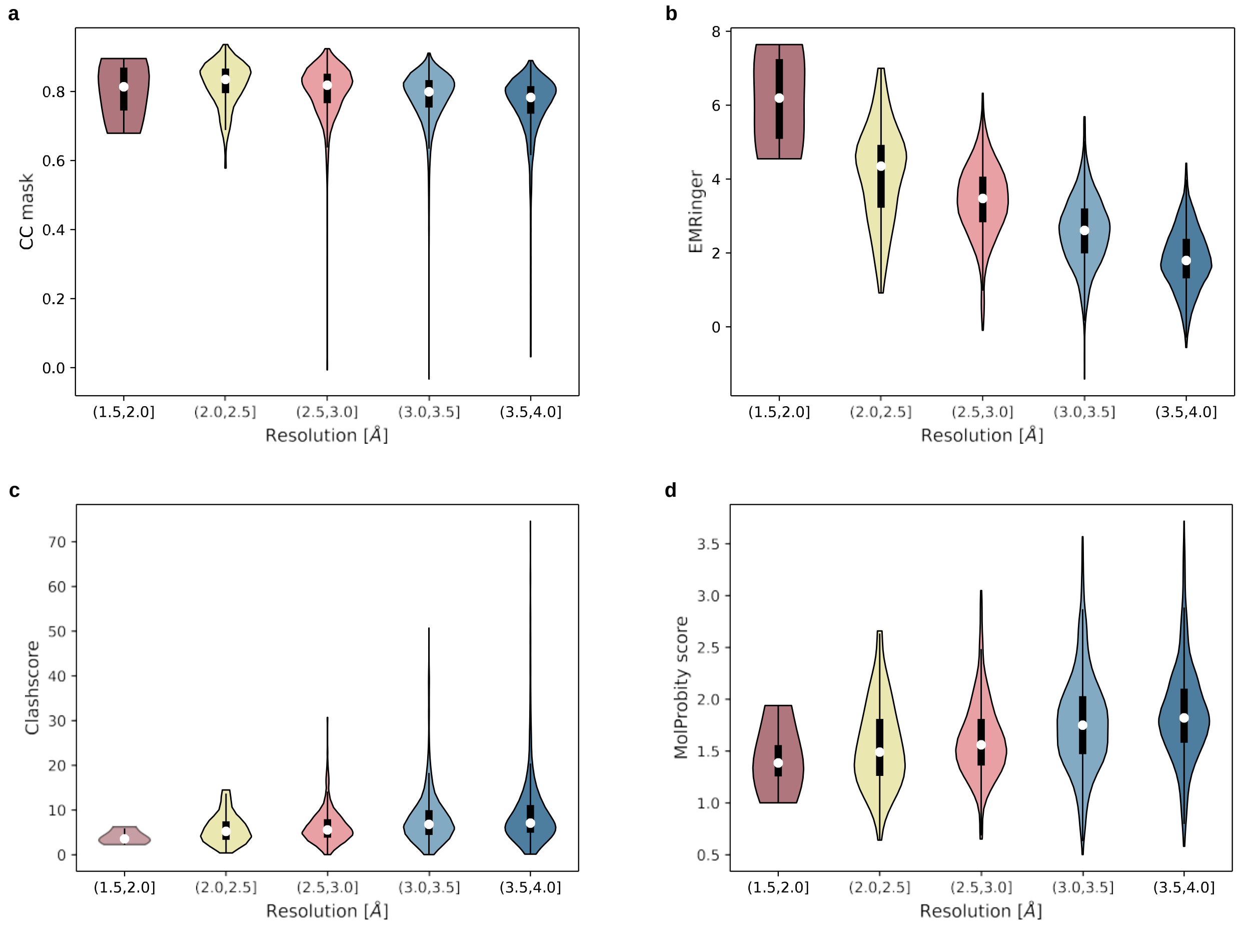

Fig B. Assessment of the quality of the cryo-EM structures deposited in the PDB database. Violin plots of (**a**) CC_{mask}, (**b**) EMRinger score, (**c**) clashscore, and (**d**) MolProbity score as a function of the resolution of the cryo-EM structures. For this analysis, 2476 structures determined by single-particle cryo-EM in the resolution range 1.78 Å to 4.00 Å and released between 2015 and 2022 were used. In the violin plots, the white circle corresponds to the median value, the black rectangle extends from the first to the third quantiles, and the thin black line represents the 95% confidence intervals.

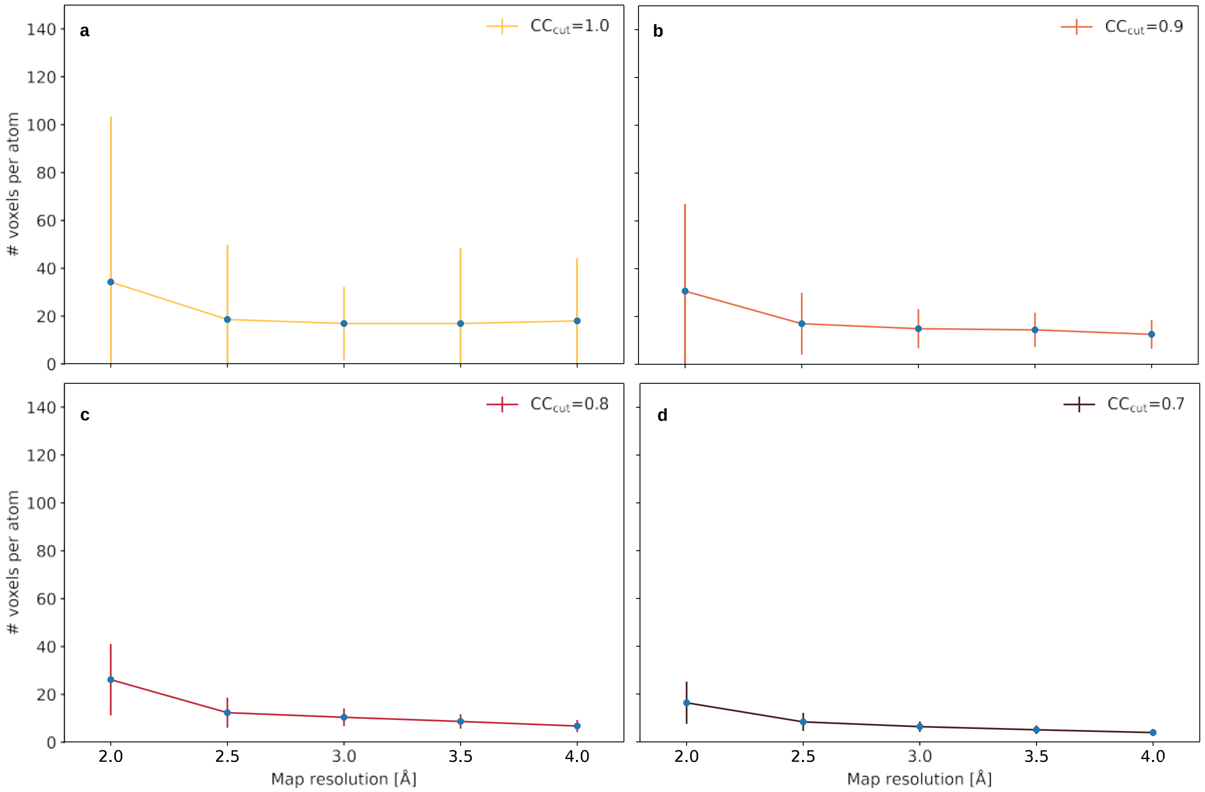

Fig C. Benchmark of the approach to remove correlated voxels. Analysis of the number of voxels per atom for 2477 structures determined by single-particle cryo-EM between 2015 and 2022 in the resolution range 1.78 Å to 4.00 Å as a function of the map resolution and voxel correlation cutoff value (CC_{cut}). Maps were separated into 5 resolution bins of size equal to 0.5 Å and ranging from 1.75 Å to 4 Å. The median values within the 5 bins are reported as blue points and the standard deviations as error bars. The reduction of the error bar at fixed resolution bin observed as CC_{cut} is decreased indicates that upon removing correlated data the number of voxels per atom across different maps of similar resolution depends less and less on the voxel size.

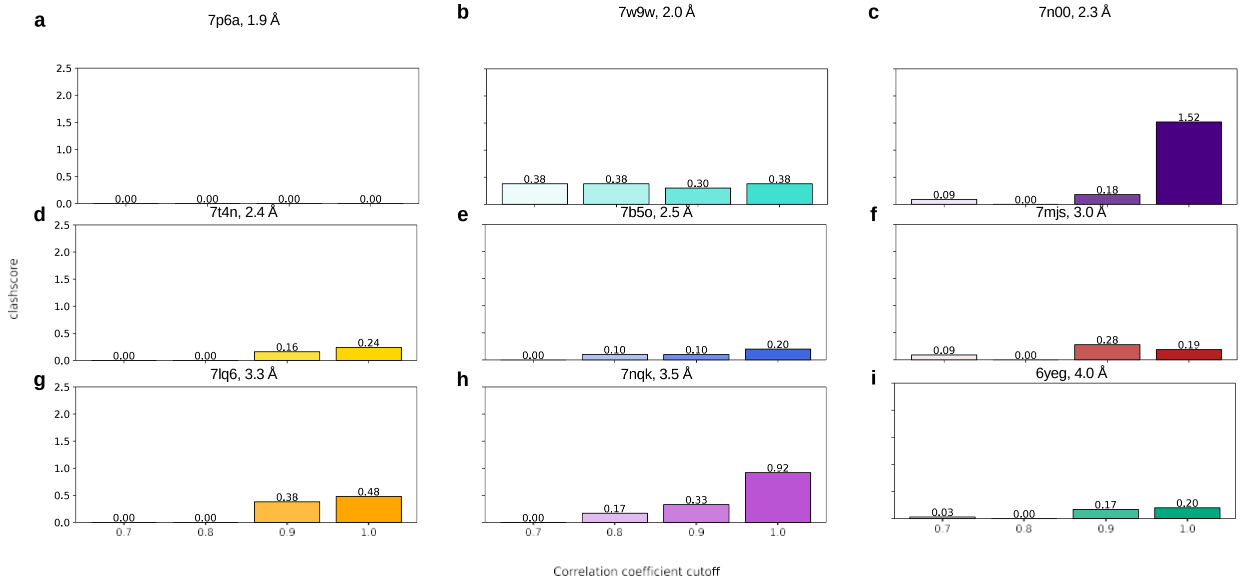

Fig D. Stereochemical quality of single-structure EMMIVox models as a function of data correlation cutoff. Comparison of clashscore for EMMIVox refined single-structure models as a function of correlation coefficient cutoff for the 9 systems in our benchmark set.

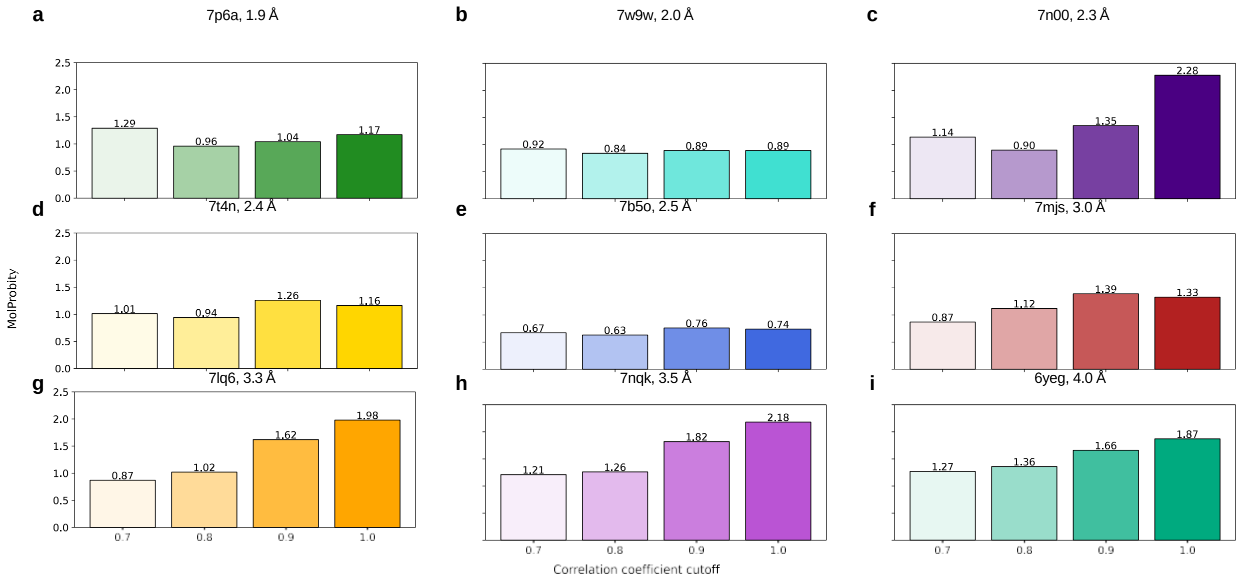

Fig E. Stereochemical quality of single-structure EMMIVox models as a function of data correlation cutoff. Comparison of MolProbity scores for EMMIVox refined single-structure models as a function of correlation coefficient cutoff for the 9 systems in our benchmark set.

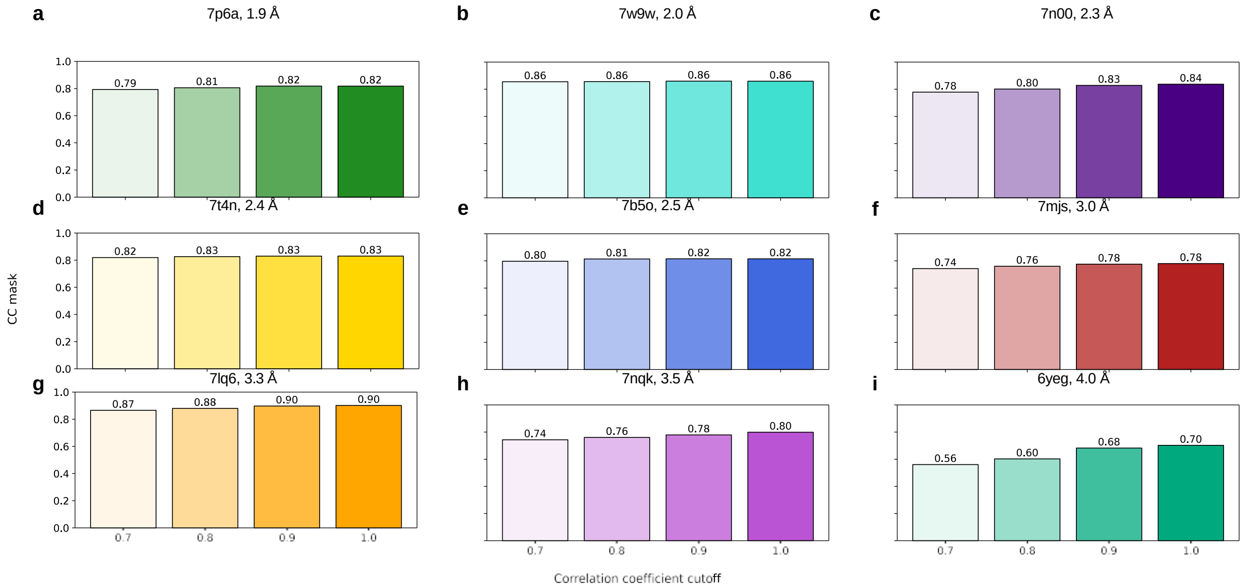

Fig F. Fit-to-data of single-structure EMMIVox models as a function of data correlation cutoff. Comparison of CC_{mask} scores for EMMIVox refined single-structure models as a function of correlation coefficient cutoff for the 9 systems in our benchmark set.

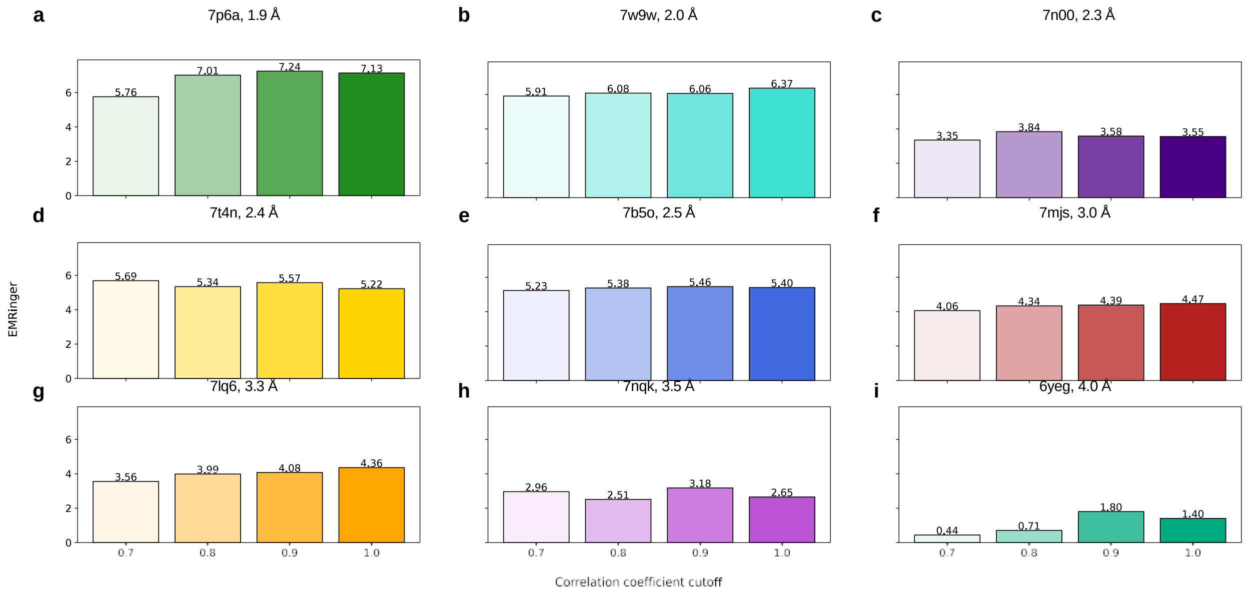

Fig G. Fit-to-data of single-structure EMMIVox models as a function of data correlation cutoff. Comparison of EMRinger scores for EMMIVox refined single-structure models as a function of correlation coefficient cutoff for the 9 systems in our benchmark set.

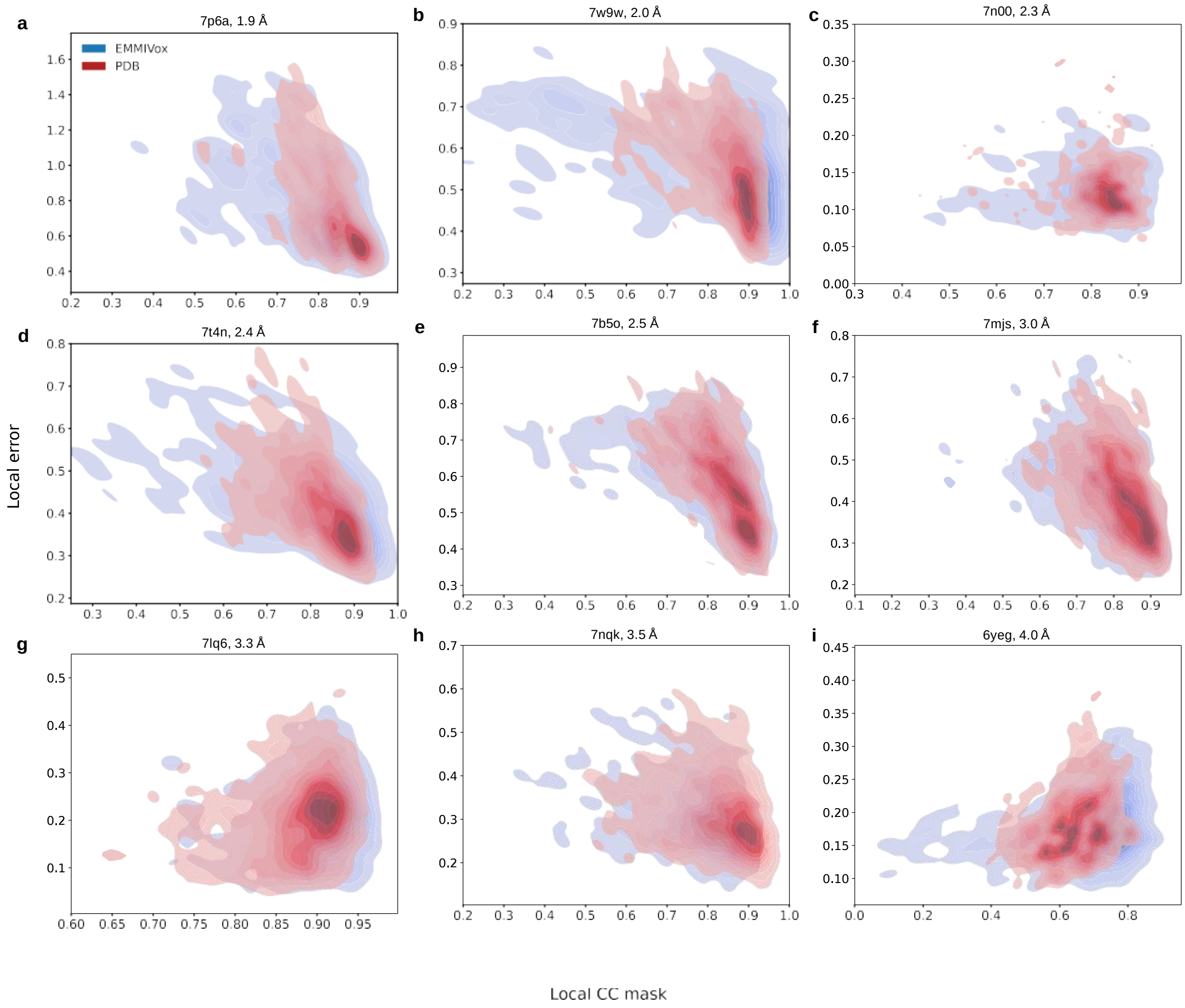

Fig H. Effect of EMMIVox noise models on model fit-to-data. Relation between individual residue fit to the experimental cryo-EM map (local CC_{mask}) and local error in the map in the proximity of each residue for the single-structure EMMIVox model (blue) and the deposited PDB (red) for the 9 systems in our benchmark set. The local error is calculated from the difference between the two cryo-EM half maps as the median value across all the voxels associated to a residue by Voronoi tessellation.

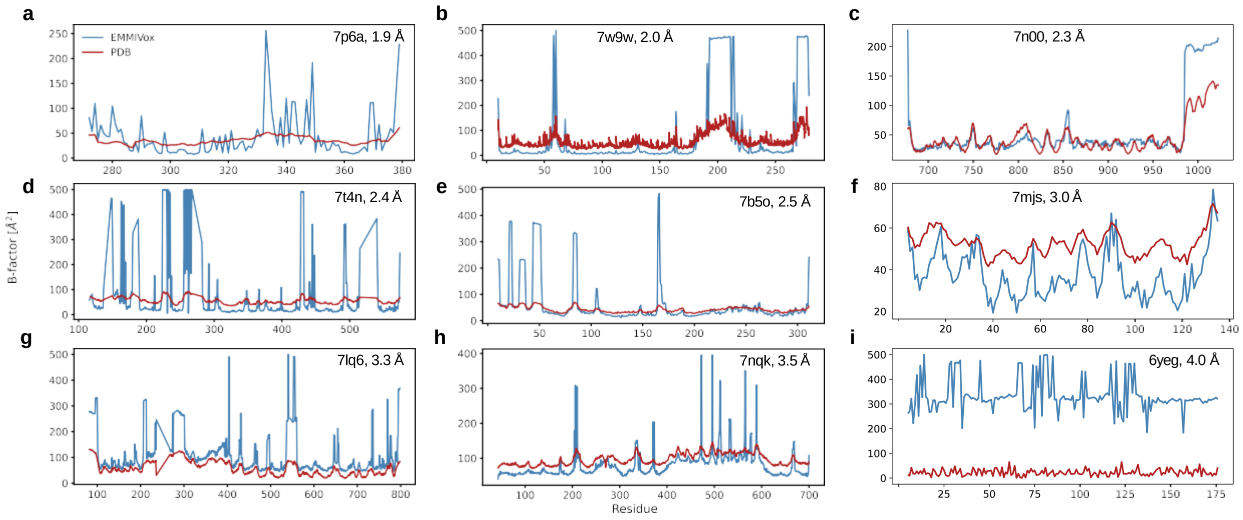

Fig I. Bayesian inference of B-factors in EMMIVox single-structure refinement. Comparison between the B-factor values of the deposited PDBs (red) and the EMMIVox single-structure models (blue) for the 9 systems in our benchmark set. Each panel compares the B-factor values for a single chain within the system and is representative of the system as a whole.

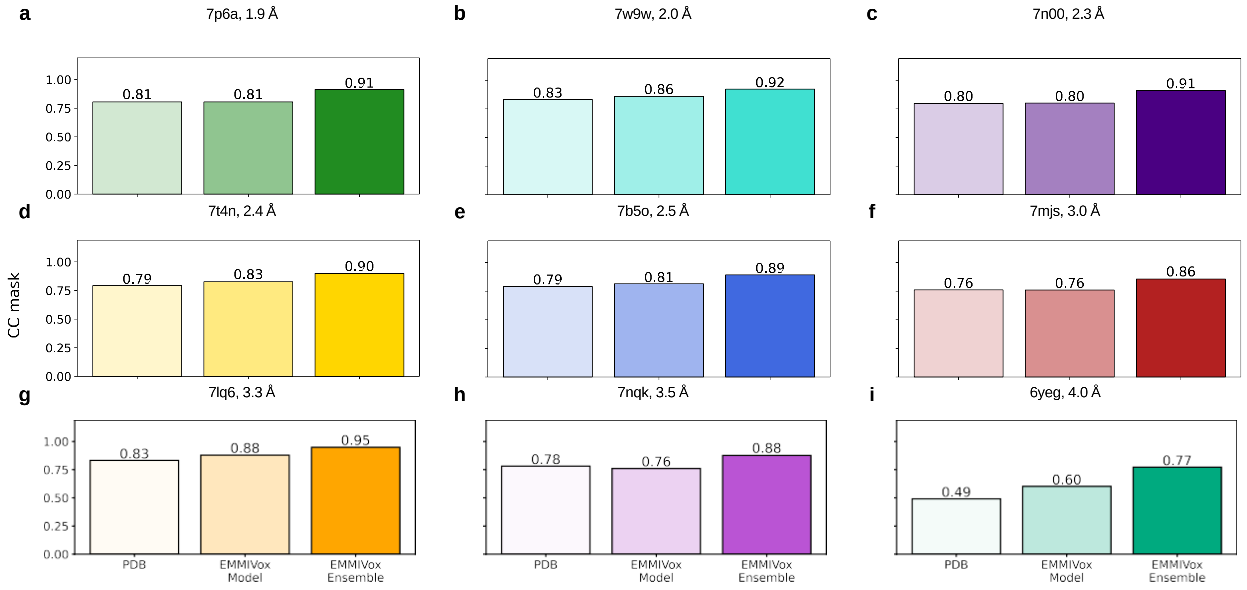

Fig J. Fit-to-data of deposited PDBs, EMMIVox single-structure models and ensembles. Comparison of the CC_{mask} scores for the deposited PDB (light color), EMMIVox single-structure model (medium color) and EMMIVox ensemble (dark color).

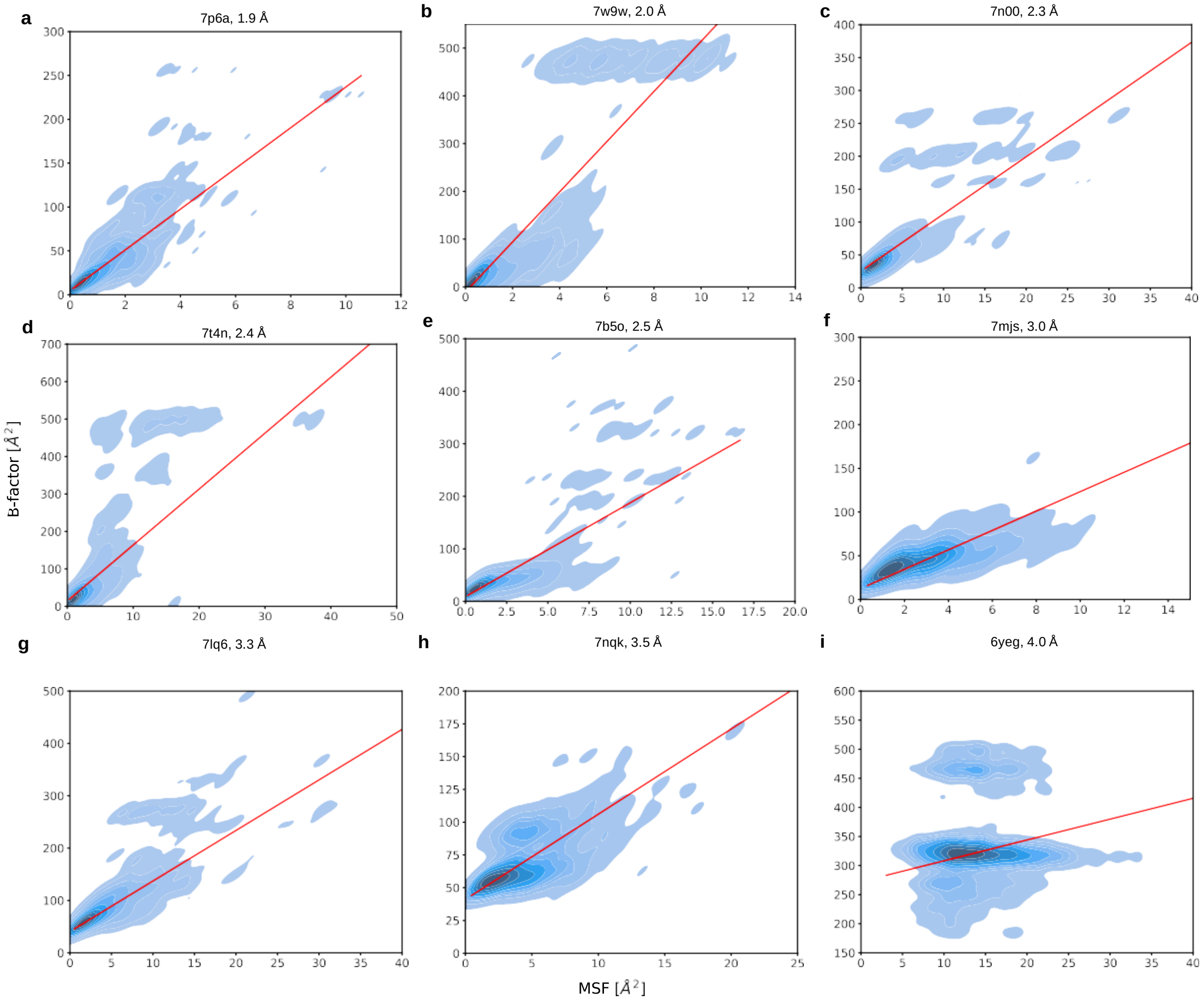

Fig K. Assessment of overfitting of data noise with conformational heterogeneity in EMMIVox structural ensembles. Relation between per residue B-factor in the single-structure EMMIVox model and residue mean square fluctuations (MSF) within the EMMIVox ensemble for the 9 systems in our benchmark set. The red line indicates a Bayesian linear fit between the two quantities.

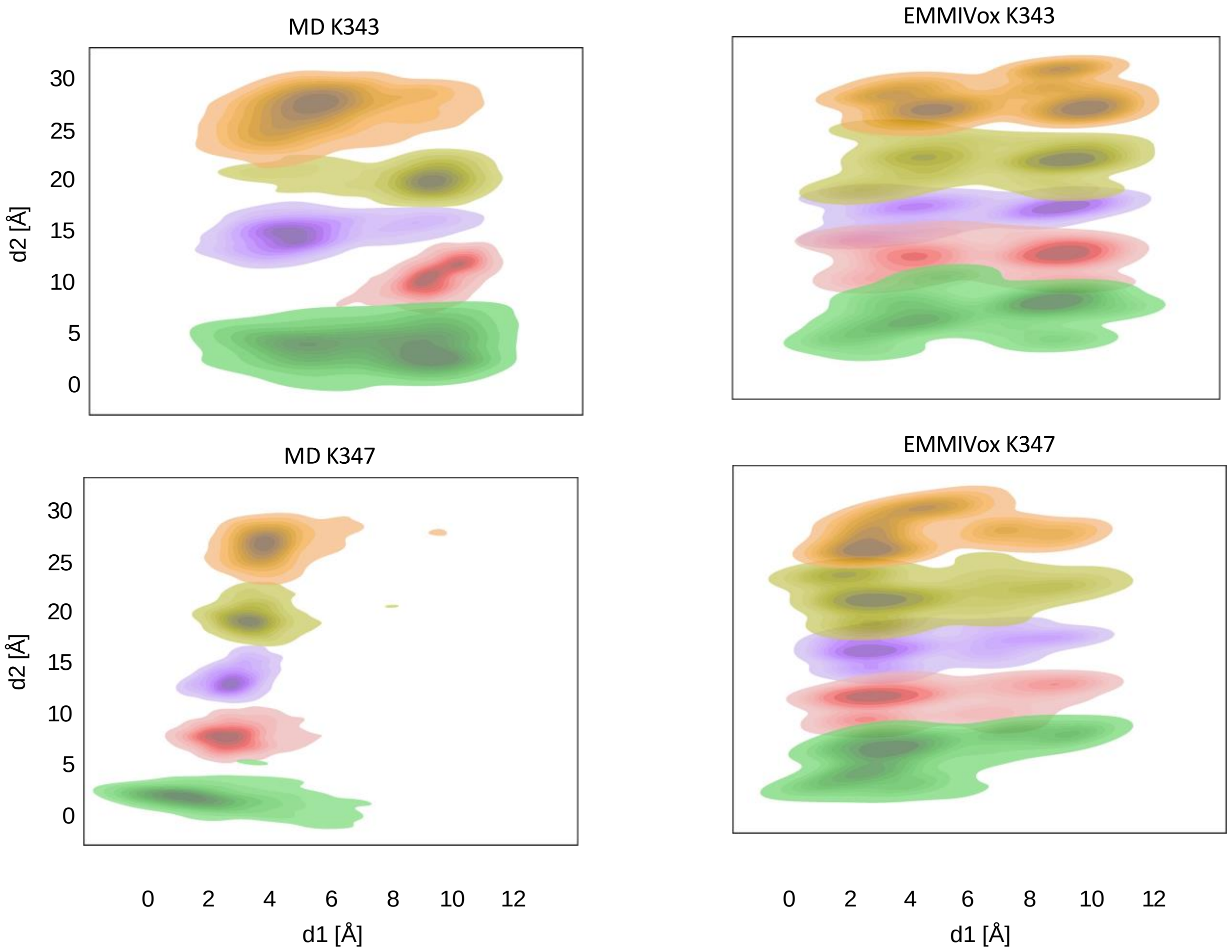

Fig L. EMMIVox sampling efficiency for the case of the GPT type 1a tau filament. Structural ensembles of residues K343 (top row) and K347 (bottom row) from standard MD simulation (left column) and EMMIVox ensembles (right column). Total simulation time for both ensembles was equal to 320 ns. In the EMMIVox ensemble, K343 occupies two distinct states with equal populations, as illustrated by the distribution of the lysine sidechain nitrogen positions projected on a plane parallel to the fibril surface. The MD ensemble for K343 shows a 2-3 split of chains occupying state 1 and state 2. Limited sampling is seen between the two states for each individual chain. K347 in the EMMIVox ensemble occupies two conformations, the major conformation found in the deposited PDB with an occupancy of 73%, and a second minor conformation, populated only by 27%. In contrast, K347 for the standard MD simulation only occupies the major conformation found in the deposited PDB and does not sample the minor conformation in the same simulation time of the EMMIVox ensemble.

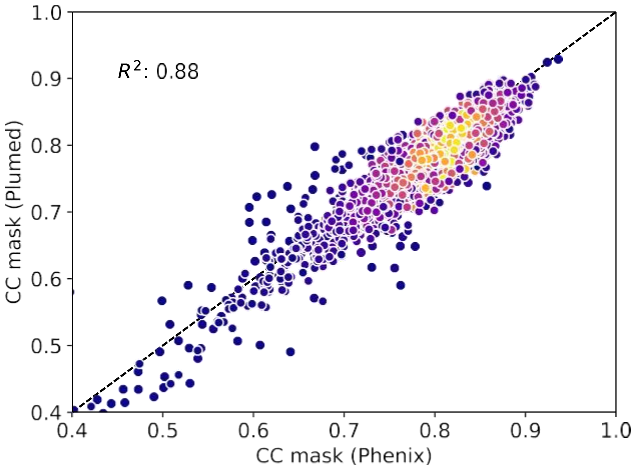

Fig M. Correlation between PLUMED and Phenix CC_{mask}. Analysis of the relation between CC_{mask} calculated with Phenix and with a custom python script for 2477 structures determined by single-particle cryo-EM between 2015 and 2022 in the resolution range 1.78 Å to 4.00 Å. Our implementation of CC_{mask} does not optimize an isotropic B-factor, unlike Phenix. Despite this difference, a strong correlation between the two CC_{mask} scores is observed across the analyzed systems. Points are colored based on local density, with yellow and blue indicating high and low densities, respectively.

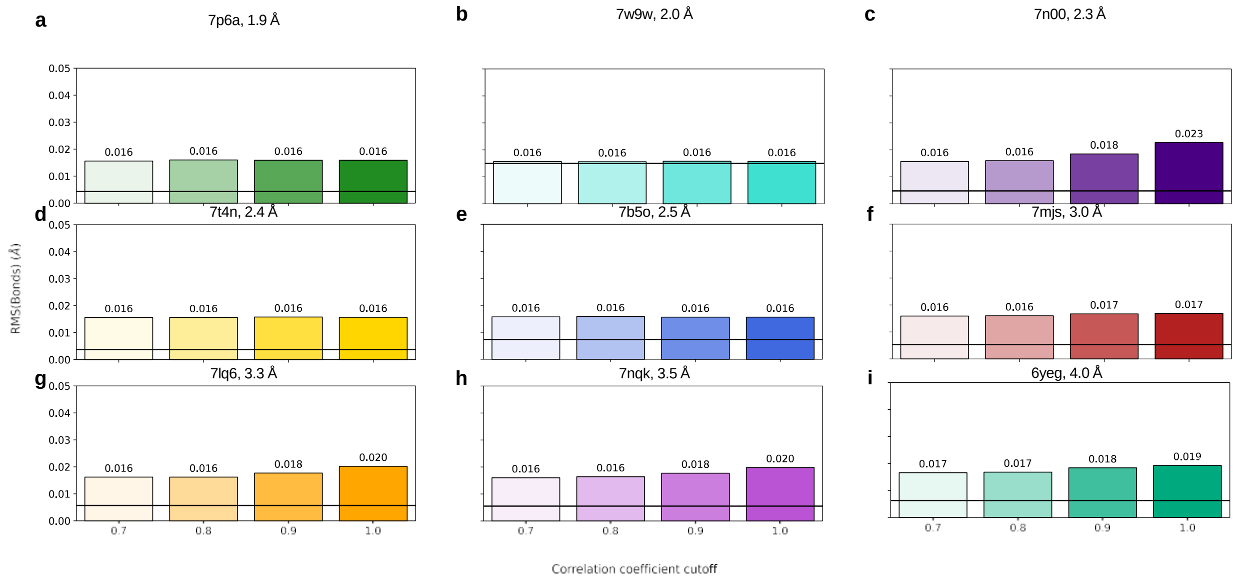

Fig N. Fit-to-data of single-structure EMMIVox models as a function of data correlation cutoff. Comparison of RMS(Bonds) values for EMMIVox refined single-structure models as a function of correlation coefficient cutoff for the 9 systems in our benchmark set. Most values fall under the 0.02 Å threshold used to define high-resolution models[1, 2]. The black line indicates the reference value in the deposited PDB.

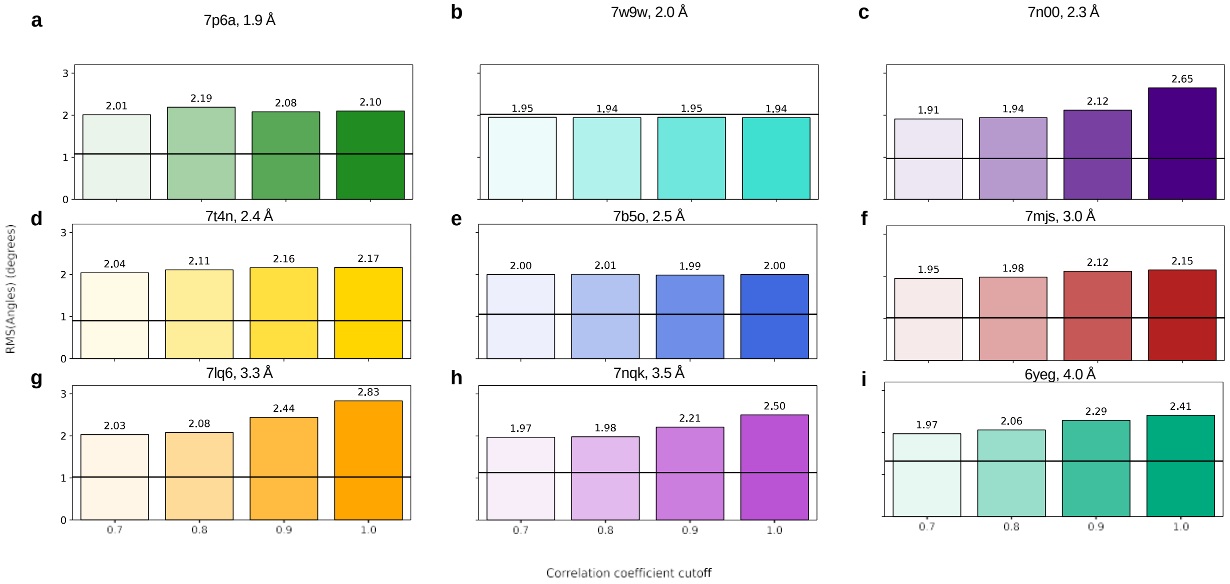

Fig O. Fit-to-data of single-structure EMMIVox models as a function of data correlation cutoff. Comparison of RMS(Angles) values for EMMIVox refined single-structure models as a function of correlation coefficient cutoff for the 9 systems in our benchmark set. Most values are within the common range of RMS(angle) values[1-3]. The black line indicates the reference value in the deposited PDB.

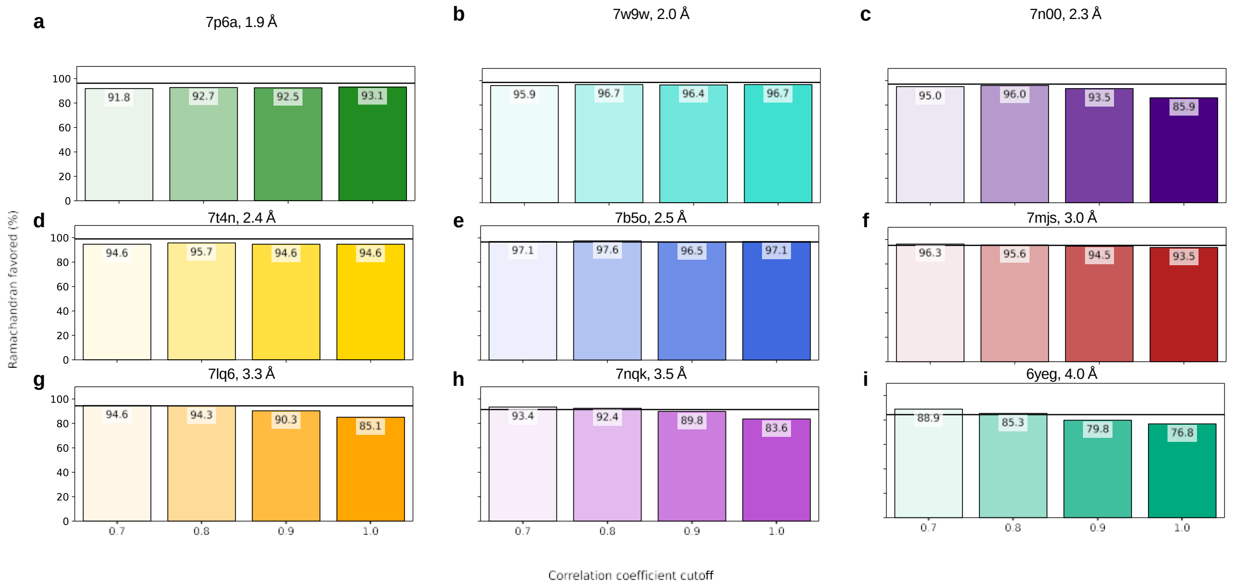

Fig P. Fit-to-data of single-structure EMMIVox models as a function of data correlation cutoff. Comparison of percentage of residues with dihedral values that fall in the favored Ramachandran region for EMMIVox refined single-structure models as a function of correlation coefficient cutoff for the 9 systems in our benchmark set. The black line indicates the reference value in the deposited PDB.

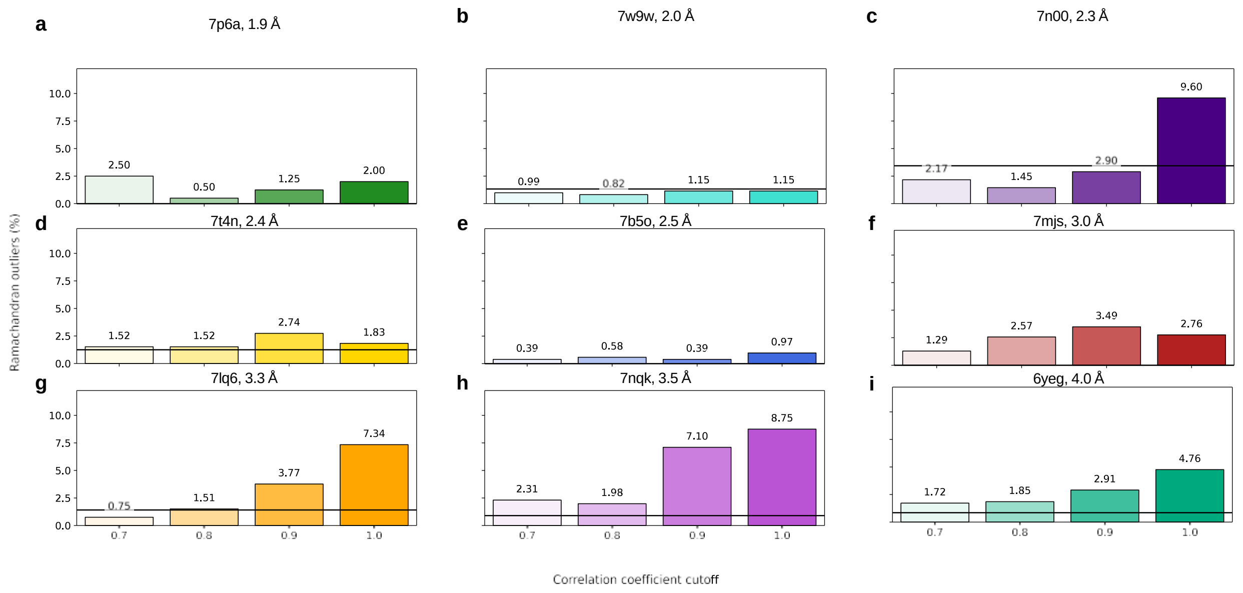

Fig Q. Fit-to-data of single-structure EMMIVox models as a function of data correlation cutoff. Comparison of percentage of residues with dihedral values that fall in the non-favored Ramachandran region for EMMIVox refined single-structure models as a function of correlation coefficient cutoff for the 9 systems in our benchmark set. The black line indicates the reference value in the deposited PDB.

Supplementary References

1. Jaskolski, M., Gilski, M., Dauter, Z. & Wlodawer, A. Stereochemical restraints revisited: how accurate are refinement targets and how much should protein structures be allowed to deviate from them? *Acta Crystallogr D Biol Crystallogr* **63**, 611 (2007).
2. Wlodawer, A. Stereochemistry and Validation of Macromolecular Structures. *Methods Mol Biol* **1607**, 595 (2017).
3. Engh, R.A. & Huber, R. Accurate Bond and Angle Parameters for X-Ray Protein-Structure Refinement. *Acta Crystallogr A* **47**, 392 (1991).

List of Legends

Table A. Parameters of the forward model to predict a density map from an atomistic model. These parameters are obtained from a 5-Gaussians fit of the electron atomic scattering factors for *s* up to 6.0 Å^{-1}. B_k parameters are expressed in Å^2.

Table B. Parameters of the forward model to predict a density map from a Martini 3 coarse grained model. B_k parameters are expressed in Å^2.

Table C. Details of the systems used in the single-structure and ensemble refinement benchmark. For each system, the first 8 columns report details about the deposited structure: PDB and EMDB ids, resolution, number of protein residues, number of protein chains, number of resolved water molecules, number of resolved lipids, number of ligands. The last 5 columns contain details of the system as prepared for EMMIVox simulations: box size, number of water molecules, number of lipids, number of buffer ions, and total number of atoms.

Fig A. The cryo-EM and cryo-ET revolutions. Number of (**a**) single-particle cryo-EM and (**b**) subtomogram averaging maps released per year and classified based on resolution. Data was obtained from the Electron Microscopy Data Bank (https://www.ebi.ac.uk/emdb/) on October 12th 2023.

Fig B. Assessment of the quality of the cryo-EM structures deposited in the PDB database. Violin plots of (**a**) CC_{mask}, (**b**) EMRinger score, (**c**) clashscore, and (**d**) MolProbity score as a function of the resolution of the cryo-EM structures. For this analysis, 2476 structures determined by single-particle cryo-EM in the resolution range 1.78 Å to 4.00 Å and released between 2015 and 2022 were used. In the violin plots, the white circle corresponds to the median value, the black rectangle extends from the first to the third quantiles, and the thin black line represents the 95% confidence intervals.

Fig C. Benchmark of the approach to remove correlated voxels. Analysis of the number of voxels per atom for 2477 structures determined by single-particle cryo-EM between 2015 and 2022 in the resolution range 1.78 Å to 4.00 Å as a function of the map resolution and voxel correlation cutoff value (CC_{cut}). Maps were separated into 5 resolution bins of size equal to 0.5 Å and ranging from 1.75 Å to 4 Å. The median values within the 5 bins are reported as blue points and the standard deviations as error bars. The reduction of the error bar at fixed resolution bin observed as CC_{cut} is decreased indicates that upon removing correlated data the number of voxels per atom across different maps of similar resolution depends less and less on the voxel size.

Fig D. Stereochemical quality of single-structure EMMIVox models as a function of data correlation cutoff. Comparison of clashscore for EMMIVox refined single-structure models as a function of correlation coefficient cutoff for the 9 systems in our benchmark set.

Fig E. Stereochemical quality of single-structure EMMIVox models as a function of data correlation cutoff. Comparison of MolProbity scores for EMMIVox refined single-structure models as a function of correlation coefficient cutoff for the 9 systems in our benchmark set.

Fig F. Fit-to-data of single-structure EMMIVox models as a function of data correlation cutoff. Comparison of CC_{mask} scores for EMMIVox refined single-structure models as a function of correlation coefficient cutoff for the 9 systems in our benchmark set.

Fig G. Fit-to-data of single-structure EMMIVox models as a function of data correlation cutoff. Comparison of EMRinger scores for EMMIVox refined single-structure models as a function of correlation coefficient cutoff for the 9 systems in our benchmark set.

Fig H. Effect of EMMIVox noise models on model fit-to-data. Relation between individual residue fit to the experimental cryo-EM map (local CC_{mask}) and local error in the map in the proximity of each residue for the single-structure EMMIVox model (blue) and the deposited PDB (red) for the 9 systems in our benchmark set. The local error is calculated from the difference between the two cryo-EM half maps as the median value across all the voxels associated to a residue by Voronoi tessellation.

Fig I. Bayesian inference of B-factors in EMMIVox single-structure refinement. Comparison between the B-factor values of the deposited PDBs (red) and the EMMIVox single-structure models (blue) for the 9 systems in our benchmark set. Each panel compares the B-factor values for a single chain within the system and is representative of the system as a whole.

Fig J. Fit-to-data of deposited PDBs, EMMIVox single-structure models and ensembles. Comparison of the CC_{mask} scores for the deposited PDB (light color), EMMIVox single-structure model (medium color) and EMMIVox ensemble (dark color).

Fig K. Assessment of overfitting of data noise with conformational heterogeneity in EMMIVox structural ensembles. Relation between per residue B-factor in the single-structure EMMIVox model and residue mean square fluctuations (MSF) within the EMMIVox ensemble for the 9 systems in our benchmark set. The red line indicates a Bayesian linear fit between the two quantities.

Fig L. EMMIVox sampling efficiency for the case of the GPT type 1a tau filament. Structural ensembles of residues K343 (top row) and K347 (bottom row) from standard MD simulation (left column) and EMMIVox ensembles (right column). Total simulation time for both ensembles was equal to 320 ns. In the EMMIVox ensemble, K343 occupies two distinct states with equal populations, as illustrated by the distribution of the lysine sidechain nitrogen positions projected on a plane parallel to the fibril surface. The MD ensemble for K343 shows a 2-3 split of chains occupying state 1 and state 2. Limited sampling is seen between the two states for each individual chain. K347 in the EMMIVox ensemble occupies two conformations, the major conformation found in the deposited PDB with an occupancy of 73%, and a second minor conformation, populated only by 27%. In contrast, K347 for the standard MD simulation only occupies the major conformation found in the deposited PDB and does not sample the minor conformation in the same simulation time of the EMMIVox ensemble.

Fig M. Correlation between PLUMED and Phenix CC_{mask}. Analysis of the relation between CC_{mask} calculated with Phenix and with a custom python script for 2477 structures determined by single-particle cryo-EM between 2015 and 2022 in the resolution range 1.78 Å to 4.00 Å. Our implementation of CC_{mask} does not optimize an isotropic B-factor, unlike Phenix. Despite this difference, a strong correlation between the two CC_{mask} scores is observed across the analyzed systems. Points are colored based on local density, with yellow and blue indicating high and low densities, respectively.

Fig N. Fit-to-data of single-structure EMMIVox models as a function of data correlation cutoff. Comparison of RMS(Bonds) values for EMMIVox refined single-structure models as a function of correlation coefficient cutoff for the 9 systems in our benchmark set. Most values fall under the 0.02 Å threshold used to define high-resolution models[1, 2]. The black line indicates the reference value in the deposited PDB.

Fig O. Fit-to-data of single-structure EMMIVox models as a function of data correlation cutoff. Comparison of RMS(Angles) values for EMMIVox refined single-structure models as a function of

correlation coefficient cutoff for the 9 systems in our benchmark set. Most values are within the common range of RMS(angle) values[1-3]. The black line indicates the reference value in the deposited PDB.

Fig P. Fit-to-data of single-structure EMMIVox models as a function of data correlation cutoff. Comparison of percentage of residues with dihedral values that fall in the favored Ramachandran region for EMMIVox refined single-structure models as a function of correlation coefficient cutoff for the 9 systems in our benchmark set. The black line indicates the reference value in the deposited PDB.

Fig Q. Fit-to-data of single-structure EMMIVox models as a function of data correlation cutoff. Comparison of percentage of residues with dihedral values that fall in the non-favored Ramachandran region for EMMIVox refined single-structure models as a function of correlation coefficient cutoff for the 9 systems in our benchmark set. The black line indicates the reference value in the deposited PDB.
